# The dualistic origin of human tumors

**DOI:** 10.1016/j.semcancer.2018.07.004

**Published:** 2018-07-21

**Authors:** Jinsong Liu

**Affiliations:** Department of Pathology, The University of Texas MD Anderson Cancer Center, 1515 Holcombe Boulevard, Houston, TX 77030-4095, United States

**Keywords:** Tumor origin, Polyploid giant cancer cells, The giant cell cycle, Blastomere, Blastomere-like, Dedifferentiation, Differentiation, Maturation arrest

## Abstract

Life starts with a zygote, which is formed by the fusion of a haploid sperm and egg. The formation of a blastomere by cleavage division (nuclear division without an increase in cell size) is the first step in embryogenesis, after the formation of the zygote. Blastomeres are responsible for reprogramming the parental genome as a new embryonic genome for generation of the pluripotent stem cells which then differentiate by Waddington’s epigenetic landscape to create a new life. Multiple authors over the past 150 years have proposed that tumors arises from development gone awry at a point within Waddington’s landscape. Recent discoveries showing that differentiated somatic cells can be reprogrammed into induced pluripotent stem cells, and that somatic cell nuclear transfer can be used to successfully clone animals, have fundamentally reshaped our understanding of tumor development and origin. Differentiated somatic cells are plastic and can be induced to dedifferentiate into pluripotent stem cells. Here, I review the evidence that suggests somatic cells may have a previously overlooked endogenous embryonic program that can be activated to dedifferentiate somatic cells into stem cells of various potencies for tumor initiation. Polyploid giant cancer cells (PGCCs) have long been observed in cancer and were thought originally to be nondividing. Contrary to this belief, recent findings show that stress-induced PGCCs divide by endoreplication, which may recapitulate the pattern of cleavage-like division in blastomeres and lead to dedifferentiation of somatic cells by a programmed process known as “the giant cell cycle”, which comprise four distinct but overlapping phases: initiation, self-renewal, termination and stability. Depending on the intensity and type of stress, different levels of dedifferentiation result in the formation of tumors of different grades of malignancy. Based on these results, I propose a unified dualistic model to demonstrate the origin of human tumors. The tenet of this model includes four points, as follows. 1. Tumors originate from a stem cell at a specific developmental hierarchy, which can be achieved by dualistic origin: dedifferentiation of the zygote formed by two haploid gametes (sexual reproduction) via the blastomere during normal development, or transformation from damaged or aged mature somatic cells via a blastomere-like embryonic program (asexual reproduction). 2. Initiation of the tumor begins with a stem cell that has uncoupled the differentiation from the proliferation program which results in stem cell maturation arrest. 3. The developmental hierarchy at which stem cells arrest determines the degree of malignancy: the more primitive the level at which stem cells arrest, the greater the likelihood of the tumor being malignant. 4. Environmental factors and intrinsic genetic or epigenetic alterations represent the risk factors or stressors that facilitate stem cell arrest and somatic cell dedifferentiation. However, they, per se, are not the driving force of tumorigenesis. Thus, the birth of a tumor can be viewed as a triad that originates from a stem cell via dedifferentiation through a blastomere or blastomere-like program, which then differentiates along Waddington’s landscape, and arrests at a developmental hierarchy. Blocking the PGCC-mediated dedifferentiation process and inducing their differentiation may represent a novel alternative approach to eliminate the tumor occurrence and therapeutic resistance.

## 1. Introduction

*“Half a century of cancer research had generated an enormous body of observations about the behavior of the disease, but there were essentially no insights into how the disease begins and progresses to its life-threatening conclusions”* [1]

Dr. Robert A. Weinberg

Understanding how cancer arises and progresses to become a life-threatening disease has been a focus of intensive study by generations of investigators. However, there is no generally accepted theory of how cancer originates and progresses. In the past 50 years, cancer biologists have focused on a model in which cancer is viewed as arising from genetic and molecular alterations in somatic cells and tumors are interpreted as clusters of fast-replicating mutant cells that survive or die according to principles of via Darwinian evolution [2–4]. This simplified working model satisfies the desire, common in this era of molecular and genetic revolution, to find simple causes for complicated diseases. However, reducing the origins of cancer to a single or a few mutations or a specific cell type is insufficient to explain the tumor represents a benign or malignant organs or pluripotent stem cell-derived teratomas that have recapitulated all three germ layers of human development. The cancer research community would benefit from a new conceptual framework that can rationally interpret the huge amount of existing molecular data generated in the past 50 years as well as the different tumor types observed by pathologists over the past 150 years. It is time to rethink “cancer biology.”

In this article, I revisit the theories of cancer arising in the context of normal development, including the century-old embryonic theory of cancer and its variants that have been proposed in recent years.I then review recent discoveries regarding induced pluripotency and the formation of stress-inducd polyploidy or polyploid giant cancer cells (PGCCs) as a potential endogenous mechanism for somatic cell de-differentiation (reprogramming), which give rise to tumors. On the basis of this new knowledge, I propose a new, unified theory of human tumors, named *the dualistic origin for human tumors*. This theory accounts for all tumors observed in oncopathology including embryonic/germ cell tumors, organ-derived tumors, and benign and malignant tumors.

I use terms found in Kumar et al.’s *Robbins and Cotran Pathologic Basis of Disease*, a textbook for first- or second-year medical students [5]. As described herein, a *tumor* is equivalent to a *neoplasia or neoplasm* and is defined as “an abnormal mass of tissue, the growth of which exceeds and is uncoordinated with that of the normal tissues, and persists in the same excessive manner after cessation of the stimuli which evoked the change” as stated by eminent pathologist R. A. Willis [6]. Tumors can be divided into embryonic or germ cell origin and an adult-organ origin. On the basis of histopathologic appearance and clinical behavior, tumors can be further divided into malignant and benign. Malignant tumors are equivalent to cancer and display a poor level of tissue differentiation, resembling the primitive tissue from which they are derived. Benign tumors display good differentiation. These terms will be used as described here to avoid any confusion that can arise from the use of *tumor* as a synonym for cancer, a practice observed in many articles in the oncology literature.

## 2. Normal development and induced dedifferentiation

The human life cycle, from zygote to adult organism, is characterized by phases of de-differentiation (or reprogramming) and differentiation [7,8]. During the first three to four days after fertilization, the zygote divides without growth in cell size to generate two blastomeres, further rounds of cell division result in four, eight, and then 16-cell blastomeres. The eight-cell blastomeres start to compact into a cellular mass with indistinguishable cell borders, a process termed *compaction*. The further division of this cell mass resembles a mulberry and is known as *morula* (the Latin word for mulberry), before forming a fluid-filled cyst, known as the blastocyst. During this period, the cells alternate between the S and M phases without the G_1_ and G_2_ phases, initially dividing synchronously but gradually moving to the asynchronous division associated with shortened telomeres, which leads to genomic chaos [9,10]. The cell mass then differentiates into a trophoectoderm and inner cell mass in this cyst, which is the first step toward an ultimate cell fate determination [11].

**Fig. 1.**
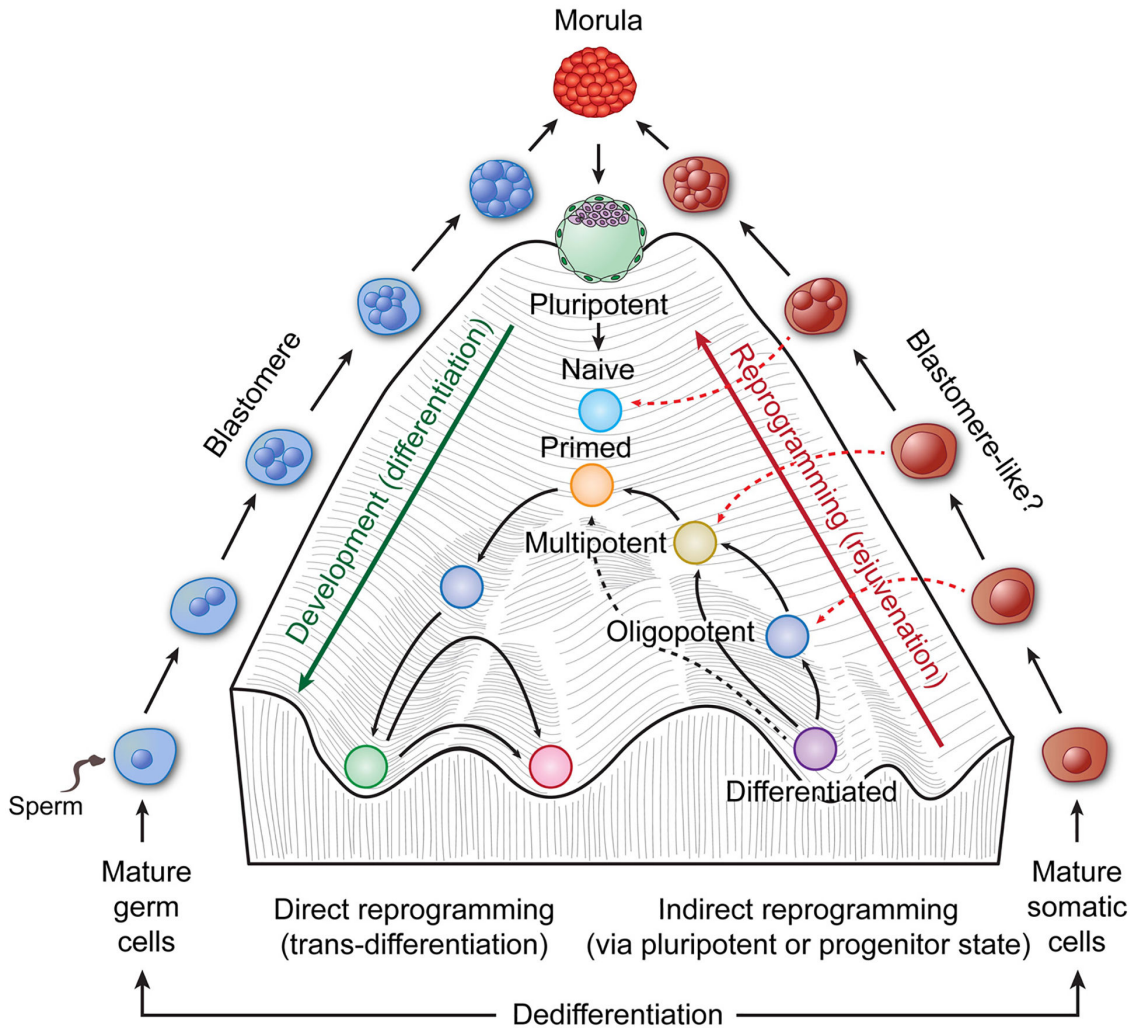
Waddington’s landscape of development (center) in the context of blastomere-mediated dedifferentiation of gametes (left), and a hypothesized blastomere-like process of dedifferentiation of somatic cells for tumor initiation (right). Following fertilization, the zygote is dedifferentiated all the way to the top of Waddington’s hill, where the compaction/morula generates a blastocyst made of trophectoderm and inner cell mass. The inner cell mass then differentiates into naïve and then primed pluripotent, multipotent, and oligopo-tent cells, and development is finally arrested at the bottom of the “canal” for a mature organism. The mature stable cells can be reprogramed via various inducing methods. The previously unknown blastomere-like pathway for dedifferentiation to generate various stem cells for tumor initiation is shown on the right side. The diagram of Waddington’s landscape and rejuvenation is adapted from these two references [12, 207].

The functions of blastomeres include erasing the epigenetic memory from the parental genome in the zygote, activating the embryonic genome, and beginning the formation of a blastocyst, which will give rise to embryoblasts and trophoblast stem cells [11]. The key function of blastomeres is to dedifferentiate the zygote formed by the mature gametes to forget the parents and to generate embryoblasts for an entire new organism together with the trophoblasts that give rise to placenta that supports its growth [11]. Differentiation from embryoblasts to adult is a highly deterministic process of tissue development, in which pluripotent cells become successively more differentiated as they roll down the “canal” to their eventual fate, a process that embryologist Conrad Waddington first termed the “epigenetic landscape” in 1950 [8,12] (Fig. 1, center). A co-culture of embryonic stem cells with trophoblast stem cells is sufficient to form a blastocyst, which is at the very top of this epigenetic landscape, capable of initiating embryogenesis in mice [13].

In 2006, it was discovered that mature somatic cells can be reprogrammed into pluripotent stem cells by four ectopically introduced transcription factors [14]. This revolutionary finding, together with the other revolutionary finding that somatic cell nuclear transfer can be used to successfully clone animals [15–17], fundamentally changed our understanding of developmental biology, regenerative medicine, and tumors. While the reprogramming in these studies was induced artificially, the data demonstrates that: (i) mature somatic cells are very plastic and can be induced into pluripotent stem cells using transcription factors or other stressors; and (ii) development can potentially be reversed (rejuvenated) in the appropriate context. These induced pluripotent stem cells can then give rise to a teratoma in vitro and *in vivo*, which is a tumor composed of mature somatic tissue (benign) or immature embryonic tissue (malignant) from one or all three germ layers, as well as other somatic cell-derived tumors including Wilms’ tumor, uroepithelial carcinoma, skin papilloma, and intestinal polyp [18]. Premature termination of reprogramming *in vivo* also leads to the development of Wilms’ tumor by the altered epigenetic landscape without genetic mutations [19]. Furthermore, it has been shown that somatic cells can be reprogrammed into pluripotent stem cells *in vivo* as a result of tissue damage and senescence [20]. These studies demonstrate that somatic-derived pluripotent stem cells can serve as the origin of teratoma, as well as somatic cell-derived tumors. This provides a novel source of origin not only for teratoma, as these tumors are generally believed to be derived from germ cells [21], but also a new source of origin for somatic cell-derived tumors. However, it remains unclear whether there is an intrinsic mechanism in somatic cells that can be induced *in vivo* to give rise to a tumor without exogenously introduced reprogramming factors.

## 3. Theories suggesting that cancer arises from development gone awry

### 3.1. Embryonic theories of cancer

Successful generation of induced pluripotent stem cells from somatic cells and the observation that these can lead to the formation of teratoma forces us to revisit the concept of cancer being embryonic origin, one of the oldest theories in pathology and cancer biology. The first two articles on the embryonic properties of cancer were published in the 19th century. Recamier (1829) [22] and Remak (1854) [23] proposed the so-called embryonic rest hypothesis of cancer origin, which was further elaborated by Durante (1874) [24] and Cohnheim (1875) [25]. Hansemann (1875) showed that cancer budding is similar to polar body extrusion in oocytes and proposed a gametogenic origin of cancer, using the word *anaplasia* (Latin for “grow backward”) to describe this process [26,27]. In addition, in 1855, Rudolf Virchow, the father of pathology and medicine, considered that connective tissues including fibroblasts and adipose tissue were full of germ cells that could give rise to cancer [28]. In 1902, embryologist John Beard developed a “trophoblast theory of cancers”, a particular interpretation of germ cells, which considered that as a result of germ cell derived trophoblasts, cancers become irresponsible [29]. These theories suggested that embryonic remnants in adult tissues or gamete-like cells could be activated to give rise to tumors.

In 1895, Wilms gave the first histological description of teratoma (called “embryoma” due to its resemblance to an embryo) and adopted the germ cell origin described first by Marchand in 1890. In this theory, the teratoma is considered to be the eggs that escape oogenesis [30]. Together with Bonnet, Marchand soon developed the blastomere theory for teratoma [30]. According this theory, the blastomere, i.e. the normal embryonic cells produced during cleavage, can escape from their normal developmental trajectory to form the teratoma, which provides an explanation as to why tissues from all three germ layers as well as these cell clusters resemble blastocyst. This was first description of the origin of teratoma. Askanazy [30] provided the first experimental proof of the embryonic origin of the teratoma. However, these concepts fell out of favor due to the lack of additional experimental support and also the shift in research efforts toward infectious diseases, influenced by the First and Second World Wars [31].

Not until almost half a century later, did Steven provide a series of experiments to generate teratoma by transplanting mouse embryo [32,33]. During the same period of time, Kleinsmith and Pierce showed that a single embryonic carcinoma cell was capable of multilineage differentiation [34]. Based on this work, Pierce proposed that tumors as caricatures of the process of tissue renewal [35]. Pierce et al. also proved that solid tumors, including squamous cell carcinoma, could differentiate into benign squamous cells [35–37]. On the basis of these data, Pierce and Sell [38,39] proposed that cancer is a developmental problem caused maturation arrest. Supporting this view, the malignant cells can be differentiated into normal tissue in mice if they are placed into a normal blastocyst [40,41].

One controversial issue is whether the targets are embryonic somatic cells or germinal cells formed via parthenogenesis, a natural form of asexual reproduction without fertilization that occurs in plants and some invertebrate animals. An unfertilized egg that can replicate itself to start embryogenesis can be a potential origin of a tumor, a view that was first proposed by Beutner in 1926 [42]. By grafting embryos with genetically impaired germ cells, Mintz et al. showed that malignant teratocarcinoma can arise from embryonal somatic cells [43]. These works provided the first experimental proof of the somatic origin of these totipotent cell-derived tumors, supporting the blastomere theory proposed by Bonnet and Marchand more than seven decades ago.

Another theory positing that cancer represents development gone wrong is the “tissue organization field theory,” or TOFT, which describes carcinogenesis as being comparable to organogenesis [44,45]. Similarities between gametogenesis and cancer development have recently been recognized, owing to the activation and expression in tumors of cancer/testis antigen and other genes normally expressed only in the germline and in embryonic cells [46]. Cancer has been proposed to be a “somatic cell pregnancy” [46,47].

The embryonic theory of cancer has also been put forward under other names, including oncogermination [48], parthenogenesis [49], and being very similar to small embryonic/epiblast-like stem cells [50]. At the experimental level, the premature termination of Yamanaka factor-induced reprogramming leads to tumor development *in vivo* [19]. Activation of a pluripotent stem cell program has been reported in bone marrow-derived cells [51,52], and germline traits have been reported in hepatoma-derived cells associated with metastasis [53]. These data demonstrate the somatic embryonic origin of human tumors, and the activation of germline traits could be as a potential result of the differentiation of embryonic somatic cells into tumors.

### 3.2. Cancer stem cell theory of cancer

In the past quarter century, a modified version of the embryonic theory of cancer has been intensively pursued that reduces the embryo to a specific stem cell type, termed “cancer stem cells”. Cancer stem cells have been proposed to be the major cause of tumor growth and drug resistance. The experiments that support this theory can be traced back to as early as 1960 based on the successful demonstration of the clonal nature of spleen colonies in transplanted bone marrow in mice [54]. This theory has been revitalized based on a study that showed that a subset of leukemia cells enriched by a combination of cell surface markers could successfully generate an entire tumor population in nude mice after a serial transplantation assay [55]. Similar experiments were conducted on a variety of solid tumors [56]. However, despite initial excitement, these subsequent studies on cancer stem cells showed that their tumor-initiating ability was not strictly dependent on the marker expression, and the tumor growth was also dependent on the mouse strain used [57–61]. In solid tumors, the cancer stem cells and the differentiated cells all grow together. In order to define the properties of cancer stem cell using serial transplantation assay, the cells in the tissue must be dissociated from their native microenvironment, resulting in changes in cell behavior and marker expression, which largely reflect their ability to adapt the mouse environment rather than the tumorigenic and differentiating properties of stem cells.

Genetic lineage tracing allows the identification of stem cells in solid tissue in situ without mechanical dissociation and has overcome some of the technical challenges associated with transplantation [62–64]. However, while lineage tracing is useful for identifying cells wired along defined developmental hierarchies, such as intestines and hair follicles, this technique has little value in high-grade tumors, such as melanoma or high-grade carcinomas from various organs, as the tumor cells in these tumors are very plastic and are poorly wired along a developmental hierarchy. Therefore, the cancer stem cell concept, although it generated much excitement and promise when it was first proposed, has been challenged in recent years [65–67].

### 3.3. Unanswered questions

Despite the long history of the embryonic theory of cancer and its variants, including the cancer stem cell theory, and the excitement about induced pluripotent stem cells and cancer stem cells, the relationship between embryogenesis and cancer stem cells remains elusive. In addition, cancer stem cells have not been conclusively linked to any specific stages of embryonic development. Given the fact that a tumor represents a disease of abnormal development, it is intuitive to assume that tumor may have simply hijacked the earliest developmental pathway for its origin. As a blastomere is at the very beginning of embryogenesis to dedifferentiate the imprinted parental genome in the zygote to create an embryonic one, it seems logical to assume that the birth of a tumor may involve a blasomere-like developmental mechanism in order to dedifferentiate somatic cells along the reverse slope of Waddington’s epigenetic landscape for tumor development (Fig. 1). However, despite the blastomere theory for teratoma being proposed by Marchard and Bonnet more than a century ago [30], it has barely been mentioned in the cancer research literature during last a half century. It was only recently, that the link between somatic-derived PGCCs and blastomeres has been made [68,109], demonstrating that blastomere-like structures and functions can also be induced from somatic cells.

## 4. Polyploidy in normal embryogenesis and polyploidy in tumors

Polyploidy, the presence of more than two sets of homologous chromosomes, can occur in both mononucleated and multinucleated cells. Polyploidy can be as a result of an endoreplication cell cycle without entering mitosis (endocycle), or entering mitosis with or without cytokinesis (endomitosis) failed at any stages of mitosis [69]. Polyploidy may play a key role in nonmalignant physiological and pathological processes. There are several excellent reviews on normal and pathologic functions of polyploidy, and readers are encouraged to refer to these for further information, particularly on the role of polyploidy in other normal developmental processes [70–73].

### 4.1. Polyploidy in normal embryogenesis

One critical topic that is not covered in previous reviews is the role of polyploidy in embryonic development. Although the blastomere stage of development is well-studied, most knowledge comes from inbreed genetically homogenous murine models [11,74]. In non-inbred genetically heterogeneous humans, the blastomere stage of development is relatively poorly understood [75], and the relevant studies are largely drawn from the field of assisted reproductive technology. Blastomere-stage embryos appear to be in a state of chaos and show marked genomic instability. In studies of fertilized eggs in vitro, approximately 60% of blastomeres grew in giant mononuclear or multi-nucleated forms [76,77]. Numerous chromosomal abnormalities (chromothripsis) were detected in normal developing human embryos [78]. The blastomerestage embryo shows increased aneuploidy, microcells, failed mitosis and cytokinesis, and endoreplication. Genetically, the interblastomere chromosome exchange may be mediated via microcells and horizontal genetic transfer [10,79]. These observations appear to be an enigma to reproductive biology on how any of us has made it this far with such a chaotic beginning [10]. Very recent findings demonstrate that there is dual-spindle formation in a zygote rather than previously believed single spindle in early mammalian embryos. These two spindles align their poles before anaphase, but the parental genomes are kept apart during the first cleavage. Multinucleated blastomeres are likely to be the result of erroneous divisions of the two spindle pole, starting from the first cleavage division [80]. The detailed process of blastomere development has been reviewed recently [74,81].

### 4.2. Polyploidy in tumors

Polyploidy in cancer has long been recognized by pathologists [26,27]. Endomitosis in tumors, a mechanism that generates PGCCs, was reported as early as 1953 [82]. Polyploidy has been reported conclusively in nearly 37% of all human tumors [83]. Mononucleated and multinucleated PGCCs are commonly observed in Barret’s esophagus, high-grade ovarian cancers and post-chemotherapy specimens [84–87] and are enriched at the invasion fronts and metastatic foci [88]. The number of PGCCs increases dramatically with the grade of malignancy in patients with breast cancer and patients with glioma [89,90]. Many recent publications report the detection of mononucleated and multinucleated PGCCs in blood specimens from cancer patients with the PGCCs appearing as macrophage-like circulating tumor cells [91,92]. It has been reported that PGCCs were visualized by fluorescence in situ hybridization, in a large majority of peripheral blood samples from patients with pancreatic adenocarcinoma [93]. In a study on mechanisms of polyploid phenotypes in breast cancer, *GINS2* was identified as the highest-ranking endoreplication-inducing gene, suggesting this molecule could be a potential biomarker for aberrant cell proliferation, and therefore a potential therapeutic target [94].

PGCCs were generally considered to be terminally differentiated senescent cells because of their inability to execute mitosis [95]. Polyploidy is commonly found in senescent fibroblasts [96]. Traditionally, progression of polyploid animal cells was thought to be limited by several intrinsic mechanisms [97–99] and a number of published findings support this view. PGCCs have been shown to inhibit tumor growth [97–99] and induce senescence [100,101], and polyploidy can suppress tumor formation in normal liver [102]. Polyploidy induced by cytokinesis failure can trigger activation of the Hippo tumor suppressor pathway [103]. These studies support the inhibitory role of polyploidy in cell proliferation and disease progression.

On the other hand, the concept that PGCCs are non-dividing has been challenged by numerous other reviews. PGCCs were shown to be capable of continuously budding and generating mononucleated cells via non-mitotic mechanisms [104]. At the beginning of this century, several investigators have independently substantiated this observation in genotoxically induced cancer cells, senescent epithelial cells or fibroblasts. Illidge et al. and Erenpreisa et al. observed the budding of PGCCs from irradiated lymphoma cell lines [86,105]; while Sundaram et al. observed a similar budding process which they termed “neosis” [106]. At approximately same time, Walen observed genomically altered daughter cells from senescent epithelial cells [107,108], demonstrating that a similar process is involved in generating transformed clones from senescent polyploidy cells. This amitotic budding has been subsequently observed by multiple other investigators including my own lab [109–115].

Polyploidy was found to facilitate the bypassing of a senescence-induced replication blockade, which effectively stimulates tumor progression [116,117]. Tetraploid cells facilitate both cancer cell survival and neoplastic transformation [95,118–120]. Gamma-irradiated tumor cells are capable of both self-renewal via PGCC formation, and PGCC de-polyploidization into paradiploid progeny [121]. Treatment of human colon cancer cells with ionizing radiation resulted in the development of viable and radiation-resistant PGCCs [114]. Docetaxel-induced PGCCs were resistant to treatment and underwent a proliferative “mitotic slippage” phase in prostate cancer [122]. Evolution of the colorectal cancer genome was accelerated via whole-genome doubling and polyploidy [123]. PGCCs with supernumerary, unstable chromosomes are likely to drive chemotherapy resistance and disease relapse across multiple types of cancers, including colon cancer, gastric cancer, and prostate cancers [124–126]. Metastatic tumors can be generated from single highly chemotherapy-resistant giant fibrosarcoma cells [110]. In addition, PGCCs have been identified in blood samples from patients with metastatic breast cancer, and higher numbers of PGCCs in blood were shown to be an independent predictor for worse progression-free survival and overall survival in these patients [91].

**Fig. 2.**
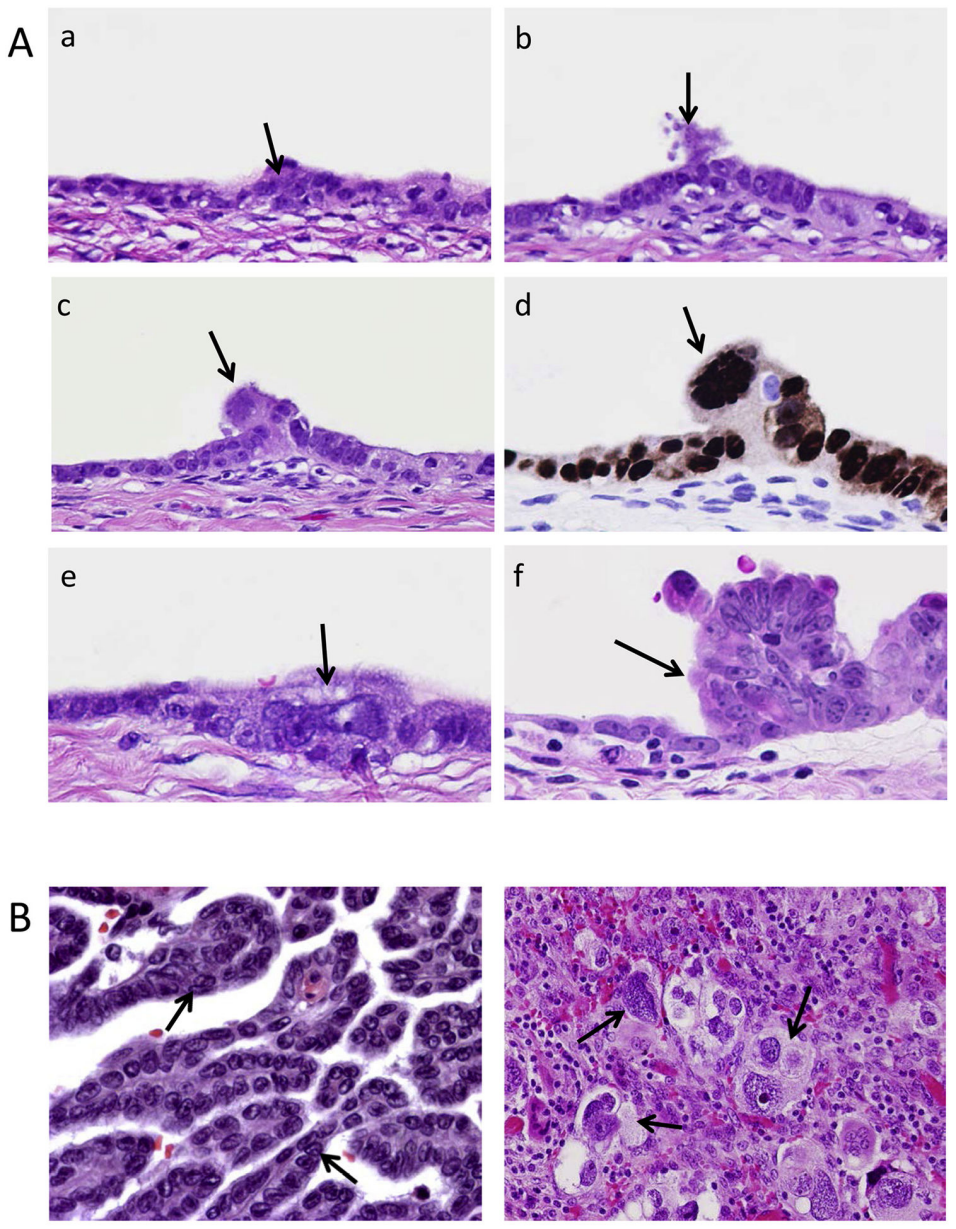
Histopathologic view of polyploid giant cells at the tumor initiation site and low- and high-grade serous carcinomas. A. Continuous sections of damaged ovarian epithelial cells with emergence of invasive high-grade serous carcinoma. A multinucleated giant cancer cell can be seen at the nidus where the invasive carcinoma (f) originates (arrow head, c, d, and e). Both in situ carcinoma and giant cancer cells are stained positive for p53. Arrows indicate the giant cells during transition from in situ carcinoma to invasive carcinoma. B. Left, an example of low-grade serous carcinoma, the nuclei of homogeneous size and absence of nuclear atypia and pleomorphism. Right, an example of a high-grade serous carcinoma with multinucleated giant cells and high nuclear atypia.

Multinucleated PGCCs are observed at the initiation site where noninvasive carcinoma transitions into invasive high-grade serous carcinoma (Fig. 2A). Multinucleated or mononucleated giant cells are observed in high-grade serous carcinoma but rarely in low-grade serous carcinoma (Fig. 2B). We demonstrated that CoCl_2_-treated ovarian cancer cells could be dedifferentiated into PGCCs with an acquired capacity to generate embryonic-like stemness and produce diploid progeny cells via budding, splitting, or viral-like bursts [109]. PGCCs near colon cancer stroma displayed budding of progeny, and the daughter cells that budded from PGCCs expressed epithelial-to-mesenchymal transition (EMT)-related proteins that are likely are associated with lymph node metastasis [127]. PGCCs have been postulated to function as stem-like cells that are capable of generating daughter progeny via endomitosis following mitotic catastrophe [106,107,128].

The role of polyploidy *in vivo* has been investigated in *Drosophila*, and these experiments suggest that polyploidy plays an important role in generating stem cells involved in wound healing and tumor initiation. Endoreplication also occurs in the injured/repairing *Drosophila* hindgut pylorus and ovarian follicle epithelium [129], which strikingly resembles the processes by which the mammalian liver fully regenerates under conditions where cell division is impaired [130]. In response to starvation of *Drosophila*, polyploidy can give arise to new stem cells in the intestine via amitosis [131]. Interestingly, genetic analyses of rab5-defective cells in *Drosophila* revealed that cooperative activation of the morphogenetic driver JNK and Yorkie (a Hippo pathway effector and YAP homolog) generates PGCCs via endoreplication [132]. The YAP activation has been shown in the formation of PGCCs and the blastomere-stage embryogenesis in humans [68,133].

Taken together, the above data suggest that PGCCs are not only capable of division but also play important roles in cancer initiation, drug resistance, and metastasis both in vitro and *in vivo* as well as in model organisms.

## 5. The giant cell cycle

**Fig. 3.**
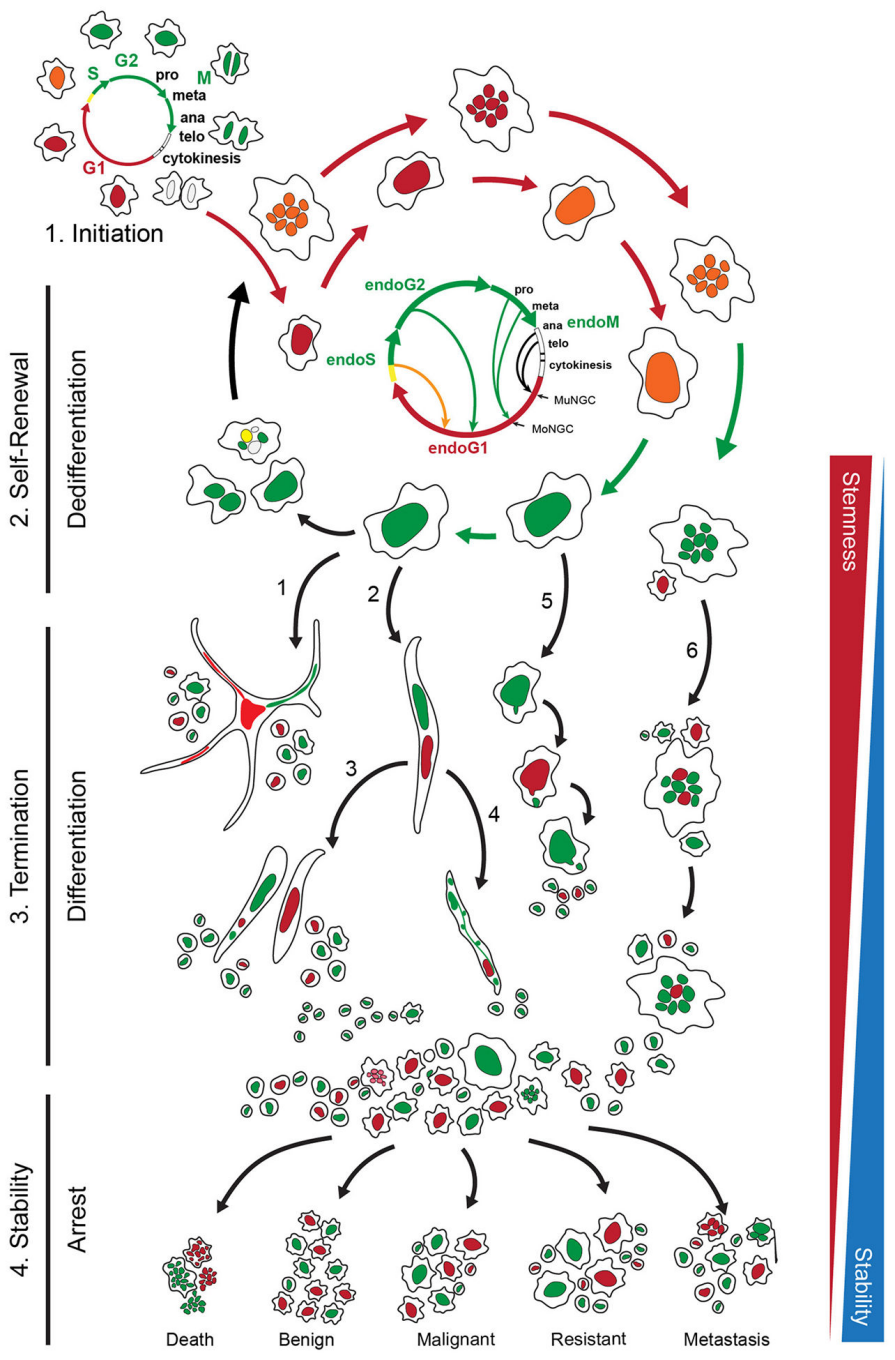
The giant cell cycle mimics blastomere division. Following initiation by either intrinsic genetic or external stresses, the somatic cell enters a self-renewal endoreplication phase and starts dedifferentiation (reprogramming). The reprogrammed cell enters a termination stage to start differentiation. During this time, giant cells use multiple modes of primitive cell division to generate diploid daughter cells, including the following: (1) horizontal genetic transfer, the DNA migrates horizontally into adjacent cells via the branch of cytoplasm and then followed by budding; (2) formation of an elongated cell with two giant nuclei followed by (3) splitting in the middle of the giant cell or (4) budding; (5) direct budding from a mononucleated giant cell; (6) direct budding from a multinucleated giant cell. During the stability phase, the differentiated cells are grown out of chaos and arrested at a specific developmental level. The dominant clones grow out of this chaos and form a visible tumor, which can behave as benign, malignant, resistant, metastasis or death (cured). The cells that have immediately budded off from the giant cells have a high level of stemness (red triangle) and gradually achieve stability during differentiation (blue triangle).

Despite of the above efforts, however, it has been unclear about how PGCCs can escape chemotherapy-induced stress in order to generate diploid progeny to support tumor initiation, drug resistance, and metastasis. In order to address this question, we used fluorescence-labeled single-cell time-lapse images to track the dynamic process of mitotic division and cell cycle progression. Using green fluorescent protein–-tracked α-tubulin, red fluorescent protein–tracked histone 2B, and a fluorescent ubiquitination cell cycle indicator (FUCCI), we temporo-spatially tracked the dynamics of cell growth and division. We observed the dynamics of mitotic spindles, and chromatin formation of giant cells and observed that these cells gave rise to mitotically competent daughter cells. We described this process as the giant cell cycle and delineated four distinct but overlapping phases: initiation, self-renewal, termination, and stability (Fig. 3) [113].

The *initiation phase* is characterized by extensive mitotic catastrophe and cell death among nearly all cells, regardless of their stage in the cell cycle. Following this massive, chemotherapy-mediated cell death, a subset of cancer cells survive and transition to tetraploidy and polyploidy. Senescence is a critical step for initiation of the giant cell cycle and is also a critical step in tissue remodeling in normal development and physiology as well as in multiple pathologic conditions [134]. The polyploid cells respond asynchronously to growth-inhibitory factors, successfully evading apoptosis/senescence. A small subset of diploid (2n) cells in the G_2_ phase display a persistent survival response evidenced by mitotic uncoupling to instigate endoreplication, disabled G_1_/S or G_2_/M checkpoint constraints, and down-regulation of mitotic mediators like Aurora A kinase, to evade programmed cell death or senescence. This allows amitotic endocycling, in which surviving cells uncouple the steps of interphase. In this amitotic mode, cells transition from endo-S back to endo-G_1_; from endo-G_2_ back to endo-G_1_; or from endo-prophase, endo-metaphase, or endo-anaphase back to endo-G_1_. We think this facilitates dual microevolutionary purposes: repetitive endo-G_1_ and endo-G_2_ phases allow for a giant cell size to resist cell death and acquire all available extracellular nutrients; and endocycling cells in endo-S generate multiple genome copies within an intact giant nucleus to resist differentiation and induce genomic reprogramming and mutational adaptation.

The *self-renewal phase* occurs as diploid cancer cells are dying and surviving cells that have transitioned to tetraploidy (4n) continue endocycling or endomitosis to produce mononucleated or multinucleated polyploid (pn) cancer cells. Some multinucleated cells undergo cyto-fission in order to generate smaller, slow-growing polyploid cells that persist through chemotherapeutic treatment via re-entering the initiation phase of amitotic endocycling. FUCCI visualization revealed that PGCC formation was via endocycling (between a G phase and S phase), which produced polytene, mononucleated or multinucleated PGCCs, all of which are truncated forms of the endoreplication cell cycle. The self-renewal stage of the giant cell cycle facilitates reprogramming and allows the somatic cells to achieve dedifferentiation and to maintain an undifferentiated state.

**Fig. 4.**
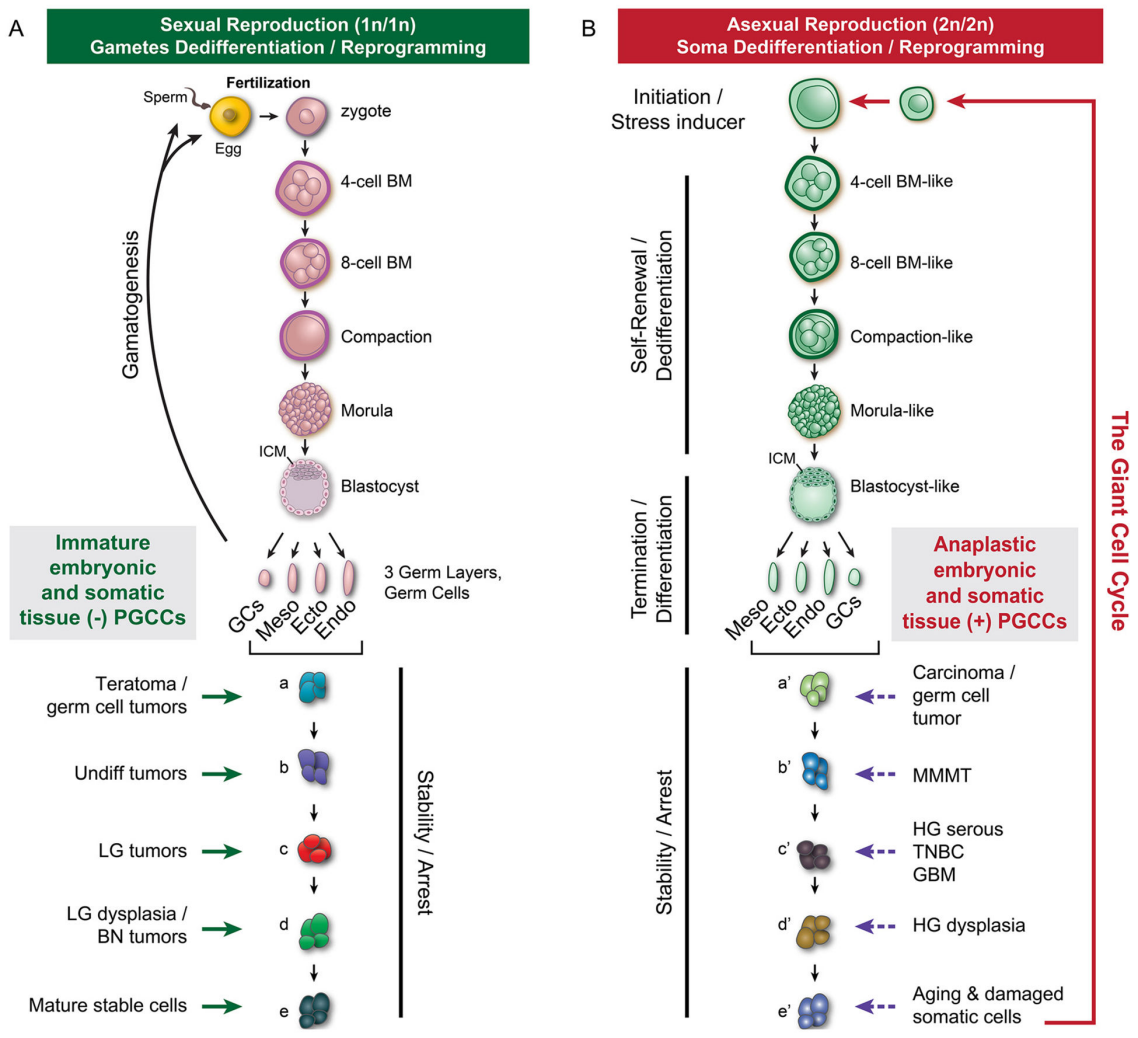
The model for dualistic stem cell origin of human tumors. Left panel (A), normal fertilization (1n/1n) triggers normal embryogenesis and gametogenesis followed by organ and adult development. The genetic and epigenetic changes lead to failure of stem cell maturation and results in their accumulation along the developmental hierarchy to give rise to a tumor. BM, blastomere; GCs, germ cells; Meso, mesoderm; Ecto, ectoderm; Endo, endoderm; ICM, inner cell mass; Undiff tumors, undifferentiated tumors; LG tumors, low grade tumor; BN tumors, benign tumors. The tumors are more common among younger people (< 50 years). Right panel (B), damaged or aged mature somatic cells and tissue undergo *de novo* transformation. The transformation process is initiated via the formation of polyploid giant cells, which can partially or completely recapitulate blastomere-like process for dedifferentiation and leads to formation of completely or partially reprogrammed stem cells and to be arrested at different levels of the developmental hierarchy. BM-like, blastomere-like; MMMT, malignant mixed Müllerian tumor; HG serous, high-grade serous ovarian carcinomas; TNBC, triple-negative breast cancers; GBM, glioblastomas; HG dysplasia, high-grade dysplasia. These tumors are more common among elderly people (> 50 years). In many tumors, both maturation arrest and dedifferentiation are simultaneously involved during their initiation and progression.

*The termination phase* occurs when some PGCCs initiate the process of depolyploidization to diploid cells, also known as genome-reductive division, using mechanisms similar to bacterial, fungal, and protozoan routes of cell reproduction. Some PGCCs spawn diploid nuclei via massive protozoan or viral-like budding to yield many smaller stem-like cells with minimal cytoplasm. In the meantime, some PGCCs form a reproductive cyst-like structure, analogous to the cysts formed by Entamoeba [135], capable of disseminating microcell progeny that may be totipotent or pluripotent, while other PGCCs employ a modified version of prokaryotic-like fission, elongating axially to produce smaller elongated polyploid progeny that can themselves begin budding diploid progeny. Still other PGCCs initiate a fungus-like sporulation, bursting and fragmenting into many diploid and aneuploid progeny; this process can also be achieved via horizontal genetic transfer, as previously described by our group in Figs. 2B and 4B [109]. It has been reported that small fragments of sheared tumor cell DNA can indiscriminately enter other cells, suggesting an evolutionarily mobile aspect of tumor-related horizontal gene transfer [136].

Finally, in *the stability phase*, spawned diploid progeny cells with novel or altered genotypes, evidenced by genomic remodeling, point mutations, or altered karyotype (i.e., chromothripsis), continue to gain cytoplasm and become more stable cells that are capable of mitosis. These spawned diploid progeny cells can go on to differentiate into a variety of cell types with variable levels of developmental potency. The resultant daughter cells with newly acquired chromosomal alterations display long-term proliferation, that can further develop into a new organ, which can behave as benign, malignant, metastatic or resistant phenotype to the original inducers [113].

In addition to endoreplication, cell fusion appears to be capable of initiating the formation of PGCCs. Cell fusion appears to be involved in generating cancer cell heterogeneity, chemoresistance, and metastasis [137,138]. In our study, treatment of cancer cells with a hypoxia mimetic led to cell fusion in approximately 10% of the cells [109]. Incomplete cytokinesis failure followed by re-fusion of small Hodgkin’s cells appears to be the mechanism by which multinucleated giant Hodgkin’s cells are generated in Hodgkin’s lymphoma [139]. The cell fusion event, together with the endoreplication described above, may represent an additional driving force to initiate formation of polyploidy and further tumor evolution.

Taken together, the experiments described here have allowed us to track the origin of resistant cancer in a stepwise programmed manner and to allow a visualization of the birth of therapy-resistant cancer cells from conventional cancer cells via formation of PGCCs [140].

## 6. The giant cell cycle as a novel mechanism for somatic cell dedifferentiation (or reprogramming)

Supporting the view that PGCCs can be dedifferentiated into cells with embryonic structure and function, we demonstrated [68] that paclitaxel-induced PGCCs are senescent. The development of single PGCCs recapitulated the development of the blastomere into morula and blastocysts. PGCCs are morphologically indistinguishable from natural blastomeres by an scanning electron microscope. The PGCCs also showed time- and location-dependent expressions of embryonic stem cell markers OCT3/4, NANOG, SOX2, and SSEA-1 and three germ layers in vitro [68]. Injection of PGCC-derived spheroids into immunodeficient mice generated mixed carcinoma and germ cell tumor and tumors of different grades [68]. In addition, hypoxic mimetic CoCl_2_-induced PGCCs were able to survive, cycle slowly, and grow back into regular diploid cells, similar to asexual division in yeast or protozoans [109]. PGCCs were capable of differentiating into adipose tissue, fibroblasts, bone, and hematopoietic cells, including neutrophils, lymphocytes, endothelial cells, and red blood cells [141–144]. Blastocyst- like strutures have been also observed in colorectal cancer cells following treatment of chemotherapy agents by Was et al. [145].

Further evidence supporting the view that PGCCs acquire embryonic stemness comes from our high-throughput proteomic profiling, which identified a set of proteins that are differentially expressed in PGCCs and regular cancer cells [146]. The functions represented by the protein set included cell cycle regulation, invasion and metastasis, stem cell generation, chromatin remodeling, and hypoxia. In addition, we showed that the addition of *Myc* oncogene, a known transcription factor involved in reprogramming could induce formation of polyploidy and convert immortalized ovarian fibroblasts to monomorphic teratoma- like tumors and induce their differentiation toward hematopoietic lineages and generation of red blood cells composed of predominantly embryonic hemoglobins [143]. Recently, additional gene transcripts interacting with *Myc* were found to be significantly over-represented in polyploid cells compared to diploid cells, suggesting that Myc-induced polyploidy may be an essential precursor to reprogramming and tumorigenesis [147].

Other evidence from different investigators also supports the concept that PGCCs acquire embryonic stemness. It has been shown that genotoxic stress can activate embryonic self-renewal in p53-defective tumor cells [148]. Poorly differentiated aggressive human tumors have an embryonic-like gene expression profile [149,150]. Further, ectopic overexpression of OCT4 in mammary epithelial cells generates tumor-initiating cells [151]. Premature termination of reprogramming leads to tumor development and epigenetic change without mutation *in vivo* [19]. Cancer cells arose from matured arrested or undifferentiated cancer stem cells via an EMT program [152,153]. The transition of somatic cells to cancer mirrors an epigenetic restriction of extra-embryonic lineages [154]. Senescence has been reported to facilitate reprogramming and cancer stem cell generation [155]. Stem cell pluripotentiality and dedifferentiation have been found across multiple solid and hematopoietic cancers [156]. In a comprehensive analysis of 11,000 tumors and 33 histotypes, dedifferentiated embryonic pheno-types were found in most high grade cancers [157]. Finally, oncogenes have been found to lead to dedifferentiation of neurons and astrocytes into glioma [158], and the introduction of core stem cell factors into mature glial cells generates glioblastoma stem-like cells [159]. Because of numerous similarities between tumors and reprogramming, cancer has been proposed as a pathologic nuclear reprogramming [160].

On the basis of the findings outlined above, we propose that the giant cell cycle represents a stress-induced endogenous mechanism for somatic cell dedifferentiation for generation of stem cells for tumor initiation. Specifically, we propose that the giant cell cycle mimics cleavage division in the embryo and signifies an atavistic, transient cellular reprogramming mechanism that induces genomic reorganization in response to various stresses [113]. Endoreplicative giant cells, in survival mode, generate progeny possessing novel, remodeled genomes that may, depending on the intensity and form of environmental stressors, adaptively dedifferentiate into an embryonic-like cancer stem cell population.

## 7. Giant cell cycle–induced chaos, germ cells, and germ cell tumors

During the normal development, chaos in blastomeres occurs during the first four days of life, a small group of cells are put aside to form primordial germ cells at the time of gastrulation after formation of the blastocyst. If the giant cell cycle recapitulates the developmental pattern and function of the blastomere then it should, theoretically, be capable of inducing a formation of different lineages, not just somatic cells but also germ cells. Our surprising finding that PGCCs are capable of generating not only three germ layers but also germ cell tumors under the influence of paclitaxel-mitotic failure [68] supports the view that the giant cell cycle recapitulates the blastomere-like program and may be sufficient to induce the formation of germ cells. The idea that cancer cells can recapitulate the most primitive growth mode for their survival has also been proposed by Erenpreisa et al., who referred to the idea as “virgin birth” [49]; Vinnistky, who proposed the “oncogermi-native theory” involving an asexual self-cloning process [48]; and Lineweaver et al, who referred to “derepression atavism” [161,162]. Activation of the primitive transcription program is detected in multiple types of solid tumors, providing molecular evidence to support this concept [163].

The chaos existing at the blastomere stage of human embryonic development must serve a critical purpose to create a new life, whether it is a normal life or a tumor. Chaos eliminates parental-pattern DNA methylation, facilitating novel genomic and epigenomic embryonic patterns. Intrinsic error-prone dual-spindle mechanisms in zygotes can facilitate the generation of multinucleation and chaos in blastomeres [80]. Chaotic aneuploidy is generated from a lack of cell cycle checkpoints, mosaicism, atypical cell division, cellular fragmentation, subchromosomal instability, micro-nucleation, and chromothripsis [81,164]. Intrinsic retrovirus or transposable elements have been shown to be common in human preimplantation embryos and pluripotent cells [165]. Microcells that drive chromothripsis have been observed in both cancer cells and embryos [140,166–168]. Endogenous retroviruses and transposable elements have been found to be activated in both blastomere-stage embryos and PGCCs [115,165], demonstrating an intriguing link between these two processes.

Polyploidy is known to be a potential driver for evolution in plant and animal ecology [169]. Blastomere-mediated chaos also explains McClintock’s observation, that cell stressors could evoke extensive genomic rearrangement to be transmitted via germlines [170–172]. Stresses, especially life-threatening stresses like X-ray irradiation and exposure to potentially lethal chemicals, can lead to genome reorganization and result in a new heritable genome for a new species, a process first described by McClintock seven decades ago [170–172]. A role in genomic chaos in cancer development has also been extensively advocated by Heng et al., [173,174].

Taken together, these findings indicate that chaos may present a robust non-deterministic mechanism to create a system as complex as a human being or a tumor through a nonlinear themodynamic. The giant cell cycle mediated chaos may represent an evolutionarily conserved fundamental mechanism in response to stresses for generating new species or rejuvenation via the formation of a germline, which is conserved through evolution from McClintock’s plants to mammals, and to tumors.

## 8. The dualistic stem cell origin of human tumors

On the basis of clinical and pathological observation as well as the experimental data described, I propose a new model to explain the origin of tumors, named “the dualistic origin of human tumors”, which includes stem cells derived from the blastomeres in normal embryogenesis and from mature somatic cells via blastomere-like mediated dedifferentiation.

The details of the dualistic model are outlined in Fig. 4, modified from Fig. 8 [68]. During sexual reproduction (Fig. 4A, left), the fertilized egg initiates cleavage division to generate blastomeres. At the 8-cell stage, the blastomere starts to compact and further divisions generate morula with the first differentiation event in the life of a human, namely blastocyst formation, in which the compacted blastomere differentiates into the trophoectoderm and the inner cell mass. Following normal development, epigenetic and genetic alterations during game-togenesis and blastomeres will be distributed into various stem cells through embryogenesis and adulthood to generate the diversity of an individual human being. The parthenogenesis of oocytes, germ cells or arrested early embryonic cells can go through full or abbreviated embryogenesis to generate all the three germ layers, including a fetiform teratoma (homunculus) resembling a malformed fetus before gastrulation [21], variable forms of monomorphic teratomas with tissue from one or two germ layers or immature teratoma if neuroectodermal tissue fails to mature after gastrulation. Tissue immaturation within teratomas or secondary transformation from mature cells generate various somatic tumors associated with teratoma.

During development and adulthood, additional genetic or epigenetic mutations or non-mutational mechanisms [175] can be introduced during organogenesis. Under environmental stresses, including chronic inflammation, virus infection, chemical carcinogens exposure, irradiation, or chemotherapy drugs, the specific stem cell prone to proliferation can be decoupled from the differentiation program to gain cell autonomy for a tumor development. As uterine implantation serves as the filter to selectively eliminate the highly genomically abnormal embryo from further development, the tumors in this group largely show immature tissue resembling that from primitive organs without identifiable mutations [18,19,176,177]. Examples of tumors belonging to this group include immature teratoma, Wilms’ tumor, ependymoma, small lymphocytic lymphoma, well-differentiated carcinomas of different organs, benign tumors, and low-grade dysplasia. As the tetraploid cells that lead to such transformation are largely transient, the tumor tissues in this group usually have minimal cytologic atypia without forming morphologically recognizable giant cells, a lack of PGCCs and gross genomic alterations.

The second source of tumor origin is achieved via the reprogramming or dedifferentiation of aged and damaged somatic cells or benign tumors following the various genetic and environmental inducers. During asexual reproduction (Fig. 4B, right), various environmental stresses together with intrinsic genetic or epigenetic alterations, can lead to relaxed cell cycle control in aged or damaged somatic cells or benign tumor cells, which in turn can lead to *de novo* dedifferentiation via the giant cell cycle. The transformation process may involve two to six endoreplication cycles to generate 4n, 8n, 16n, 32n, 64n cells respectively similar to cleavage division to generate stem cells with variable developmental potency. The higher the number of the endoreplication cycle the more of infidelity of DNA replication, the more complete the dedifferentiation, which can be up to the morulae stage embryo for teratomas. Similar to stem cell arrest from normal embryogenesis, the level of malignancy corresponds to the level of developmental hierarchy that these stem cells arrest. The more primitive the stage of developmental hierarchy at which the arrest occurs, the more malignant the tumor is likely to be.

Unlike tumors generated from sexual reproduction, there is no uterine implantation involved in tumors generated from asexual reproduction. Depending on the pre-existing epigenetic and genetic alterations or organismal structures, tumors generated along this asexual reproduction pathway display high nuclear pleomorphism with variable numbers of PGCCs. These tumors usually occur among elderly patients and have numerous genomic and epigenetic alterations at the chromosomal levels. Examples include mixed carcinoma and germ cell tumors, malignant mixed Müllerian tumor, high-grade ovarian serous carcinoma, triple-negative breast cancer, glioblastoma, anaplastic lymphoma, and high-grade dysplasia, and post-chemotherapy or radiation therapy-treated resistant tumors.

This dualistic model has incorporated multiple elements from early models proposed by multiple investigators. The blastomere origin for teratomas was proposed by Marchand and Bonnet more than a century ago [30], maturation arrest was proposed by Pierce [178], although dedifferentiation was considered as unnecessary in his model. A model to explain the malignancy based on stem cell potency was also proposed by Biava and Bonsignorio [179]. Asexual clonal reproduction was proposed by Vinnisky and was further refined by Erenpreisa et al. to incorporate polyploidy in the process [48,49]. However, tumors derived from normal development via sexual reproduction, and the concept of maturation arrest generating different malignant and benign tumors along developmental hierarchies was not considered in their model. As the giant cell cycle recapitulated many features of the life cycle of single celled organism like Amoeba [135]. This model also incorporates the atavistic model from single celled organisms to multicellular organisms via activation of ancestral gene networks [161,163,180]. Thus, the dualistic model provides a unified model for all human tumors. The detailed similarities and differences between the stem cells from blastomere and blastomere-like origins are shown in Table 1.

However, it must be emphasized that the type of tumor generated via sexual and asexual pathways is most likely dependent on the preexisting genetic change in the genome, inducers, and the micro-environments. For example, BRCA1 and BRCA2 germline mutations are always associated with high grade carcinomas in the breast and ovary [181]; Reprogramming *in vivo* using transcription factors from normal healthy somatic cells, presumably without previous tissue damage in these animals, can generate Wilms’ tumors, uroepthelial carcinoma, and skin papilloma [18], similarly to those generated from sexual reproduction. In many tumors, both sexual and asexual mechanisms are used in their initiation and progression.

## 9. Relationship between the dualistic model and other models of cancer origin

The dualistic stem cell model provides rational explanations for several existing models of the origins of cancer: the somatic mutation model, the multistep progression model, and the cancer stem cell model.

### 9.1. Somatic mutation model

In the somatic mutation model, cancer arises from an uncontrolled proliferation of cells due to oncogene activation or loss of tumor suppressor genes from quiescent somatic cells. This gene-centric view of cancer development has recently been challenged by the wide range in the number of mutations per tumor identified in the pan-TCGA project [44,45]. Some tumors acquired thousands of mutations, including mega-genomic deletions and duplications, while others lack any mutations at all. Furthermore, many mutations have been reported in normal tissues, arguing against the driver role of somatic mutations in tumor initations.

In the dualistic model proposed here, the mutations, per se, are not required for development of cancer as reprogramming can be achieved via activation of normal embryonic transcription factors. The genetic mutations, however, particularly in inherited cancers, like p53 in Li-Fraumeni syndrome, retinoblastoma genes in retinal cancer, or BRCA genes in familial breast cancer, can prime the somatic cells for dedifferentiation. Other mutations, such as *KRAS* or *BRAF*, may uncouple proliferation from differentiation and can lead to inhibition of stem cell maturation. The other genetic changes may directly or indirectly affect the epigenetic mediated programming and reporgramming and lead to stem cell maturation arrest [182].

### 9.2. Multistep progression model

Another commonly described model of cancer origin is the progression from benign to malignant tumors via the accumulation of mutations. In the dualistic stem cell model, benign and malignant tumors can be explained by the arrest of stem cells at different developmental hierarchies. Depending on the severity of the intrinsic genetic or external insults and the microenvironment in which particular somatic cells reside, dedifferentiation may proceed toward a primitive stage of embryogenesis, resulting in a malignant tumor; or dedifferentiate to a less primitive stage of embryogenesis, resulting in a benign tumor. Supporting this interpretation, it has been reported that many malignant tumors, including tumors of the colon, breast, and pancreas, are initiated via a single catastrophic event. This phenomenon is called the “big bang” model of development [183–185], most likely due to high number of endoreplication cell cycles required to achieve the higher level of dedifferentiation. These cells then arrest at the primitive stage of the developmental hierarchy for these organs to grow into a malignant organ. On the other hand, some benign tumors, including colon polyps, which may be low level of dedifferentiation by low number of endoreplicaton cell cycle. However, benign tumor can be further dedifferentiated by additional endorereplication cell cycles in a stepwise manner to be transformed either into invasive cancer or completely regress.

### 9.3. Cancer stem cell model

The concept of cancer stem cells can be neatly explained in the dualistic model. Batlle and Clevers proposed that cancer stem cell hierarchies in many types of cancer are not fixed and that stochastic or chaotic tissue micro-environmental contexts might yield interconversion among cancer stem cells and non-stem cancer cells [62,186].

In the dualistic model presented here, some cancer stem cells arise during normal embryonic and adult development, while others arise from PGCCs during *de novo* transformation. The immediately budded daughter cells from PGCCs show activated expression of cancer stem cell markers, including ALDH1a, CD133, and CD44 [68,109,115,187], indicating that at least some cancer stem cells may be daughter cells that have immediately budded from PGCCs. However, it is difficult to compare the stem cells in different experimental systems. For example, hair-follicle stem cells are clearly different from stem cells in highly malignant tumors like glioblastoma and high-grade serous carcinoma, which are very plastic and highly prone to dedifferentiation. PGCCs provide a stable experimental system to track these cells expressing these markers, which should help further clarify the relationship between marker-defined cancer stem cells and PGCCs.

## 10. The relationship between dualistic stem cell model and other cancer phenomena

In addition to the several popular cancer origin models described above, there are several cancer-related phenomena that are currently under active investigation. The dualistic stem cell model also provides rational explanations for several of these phenomena.

### 10.1. Epithelial-to-mesenchymal transition

Epithelial to mesenchymal transition (EMT) plays an important role in multiple stages of development. Improper balance between epithelial-to-mesenchymal ratios leads to tissue maturation arrest and tumor development, either sarcoma or carcinoma. In recent years, EMT has been intensively studied as a potential mechanism for invasion and metastasis because of the increased mobility of mesenchymal cells [152,188]. However, recent data suggest that EMT may not be required for metastasis, at least in some cancer models [189,190]. EMT promoting transcription factor Snail has been shown to be capable of actviating expression of the filament forming protein septin-6, resulting in the midboy persistence, abscission failure, and multinucleation of tumor cells, and tissue stiffening, which can promote the genomic instability and cancer progression [191]. EMT is involved in multiple stages of human development, and the role of EMT is clearly different in the development of tumors arising from stem cells arrested at a high level of development (malignant) and tumors arising from stem cells arrested at a late stage of development (benign). Therefore, the role of EMT in tumor metastasis must be interpreted within the context of development with great caution: A highly malignant tumor may not need to undergo EMT for dissemination; Similarly, a benign tumor may not possess sufficient stemness to be able to spread to distant sites regardless of whether it has acquired a mesenchymal phenotype or not. In addition, therapeutic stress may trigger differentiation toward benign lineages via EMT rather than promoting the proliferation and migration associated with the metastatic phenotype [144].

### 10.2. Senescence, immortalization, and transformation

The dualistic model also provides a rational explanation for the long-known paradox that senescence is associated with stemness and also an explanation for the senescent-associated secretory phenotype [134, 192]. Senescence has been shown to be a major source of cancer stemness via reprogramming [155]. In addition, senescence is a prerequisite for cells to achieve immortalization or transformation from primary cells: crisis usually follows senescence and is associated with massive karyotypic changes, among them, that an immortalized or transformed cell can grow indefinitely [193,194]. Shortening of telomeres leads to a crisis, and cancer development is associated with telomere stability through re-expression of telomerase. Following the anti-telomerase therapy, the cancer cells re-enter crisis and grow into fit, stable clones expressing alternative lengthening of telomerase pathways [195,196]. All of these phenomena can be explained by the giant cell cycle mediated dedifferentiation and reprogramming.

### 10.3. Warburg effect

Warburg et al. observed that tumors use aerobic glycolysis to generate lactate from glucose in the presence of O_2_ [197]. Thousands of papers over the past ten years have delineated the underlying mechanisms and functions [198]. Cancer has been considered as a mitochondria metabolic disease [199]. We have reported that embryonic hemoglobin and embryonic red blood cells can be generated from fibroblasts in the presence of hypoxic mimetic CoCl_2_ [143], and the embryonic hemoglobins can also be observed in high-grade cancer cells [143]. Loss of CSL, a component in Notch signaling, unlocks the hypoxic response and allows cancer cells to acquire a PGCC phenotype [200]. The ability to generate embryonic hemoglobins for O_2_ also explains the failure of some clinical trials of angiogenic therapy and resistance to such therapy: anti-angiogenetic therapies can only stabilize particular subsets of cancers, as angiogenesis occurs late in development.

### 10.4. Clinical and pathologic features of patients’ tumors

Two types of tumors that have long been observed by pathologists are (i) immature tumors lacking PGCCs and nuclear atypia or heterogeneity and (ii) tumors associated with anaplasia (backward growth). Tumors in the first group show overgrowth corresponding to the tissue in early embryonic and adult tissue development and lack of PGCCs without nuclear atypia or heterogeneity. Examples include immature teratoma, Wilms tumor, low-grade serous carcinoma, small lymphocytic leukemia, and cystadenoma. Tumors in second group are derived from transformation of dedifferentiation from somatic cells via a blastomere-like mechanisms. These tumors show multiple PGCCs with marked heterogeneity and anaplasia. Examples include dedifferentiated liposarcoma, high-grade ovarian serous carcinoma, triple-negative breast cancer, and glioblastoma. In the female reproductive system, type I epithelial tumors belong to the first group from sexual reproduction, and type II tumors belong to the second group from asexual reproduction [201]. The additional details can be found in Table 1.

## 11. Therapeutic implications of the dualistic model

Definition of the giant cell cycle not only helps us to understand tumor initiation, acquisition of resistance to therapy, and disease relapse, but is also offers several previously unappreciated targets for cancer therapy.

First of all, senescence represents the first step in the initiation of the giant cell cycle. Thus, anti-inflammatory agents that induce senescence or the senescence-associated secretory phenotype could be employed as a tumor-prevention strategy. Therapeutic targeting of senes-cent cells, or pro-senescent therapy, may induce a tumorigenic reversion or anti-malignant tissue microenvironment. The role of senescence in the giant cell cycle also provides a rational explanation for the long-established chemo-preventive effect of aspirin and also suggests the use of anti-inflammatory agents to prevent formation of polyploidy to enhance the therapeutic effect and prevent disease relapse [202].

Second, self-renewal and termination stages of giant cells are associated with primitive division. Drugs that are antibacterial, antifungal, or anti-protozoan may be effective [126]. In addition, a recent study that utilized chemical inhibitors against centromere-associated protein CENP-E and kinesin-related protein Eg5 to study cell fates following mitotic slippage may have implications for anti-microtubule drug treatment [203].

Third, human embryogenesis and fetal development are immunoprivileged; similarly, the giant cell cycle recapitulates embryonic reprogramming, which is also immune privileged. Therefore, targeting PGCCs may require combinations of immunomodulatory drugs. In addition, early embryo-like structures derived from PGCCs may represent a potential tumor vaccine. Supporting this view, induced pluripotent stem cells have been reported to elicit anti-tumor response [204].

Finally, successful treatment of cancer must be directed toward blocking the different stages of the giant cell cycle and differentiating arrested malignant tumors toward becoming benign tumors, the so-called “differentiation therapy”, first advocated by Pierce et al. in 1970 [205]. Differentiation therapy has been successfully used in treatment of acute myelogenous leukemia [206], although it has had only limited success with solid tumors. The PGCCs offer new targets for differentiation therapy, and differentiation agents should be induced concurrently with chemotherapy, not afterwards.

## 12. Conclusion

Looking back on the history of cancer research, the understanding of the origins of cancer has taken multiple turns over the past two centuries. The embryonic theory of cancer, based on the intuition of pathologists, was first proposed nearly 150 years ago. At the beginning of the 20th century, two parallel theories of cancer origin, the somatic mutation theory and the cancer stem cell theory, were proposed [186]. In the past 50 years, the somatic mutation theory has been dominant, and the cancer stem theory has fluctuated in popularity. In the past two decades, the understanding of cancer has moved from the view that cancer is caused by a single or a few genes to the current concept of it being an enormous, genetically heterogeneous collection of diseases caused by differential mutations [2–4], that require individualized therapy based on specific mutations.

In my view, cancers should be viewed as a group of defined diseases arrested at different stages of development from stem cells generated via sexual and asexual reproduction. Efforts should be directed toward targeting the giant cell cycle and to make the arrested stem cells and to differentiate again into mature benign cells in addition to cytotoxic study and immunotherapy.

It has been about half a century since Presdient Nixon launched War on cancer. Billions of dollars have been invested in fighting this disease but no insight has been gained on the initation and progresssion of this disease. Maybe it is time for cancer researchers to re-examine some of the established concepts in cancer biology and make a new conceptual turn again.

## Figures and Tables

**Table 1:** Comparison of Human Blastomere and Blastomere-like Program for Dedifferentiation in Dualistic Model.

	Blastomere	Blastomere-like
Mode of reproduction	Sexual	Asexual
Starting cells	Haploid, genomic imprinted sperm & egg (1n & 1n)	Specialized diploid somatic cells (2n)
Initiation	Cell fusion (2n,2c)	Endoreplication or cell fusion Tetraploid, polyploidy (≥4n,4c)
Potency	Totipotent zygote	Specialized somatic cells
Division	Nuclear Cleavage	The giant cell cycle (≥4n,4c); Endoreplication, amitosis [113]
Microenvironment	Zona pellucida	Somatic environment
Nuclear to cytoplasmic ratio	Progressive increase	Progressive increase [113]
Genomic instability (chaos)	Failed/asymmetrical cytokinesis, microcell, nuclear fusion, aneuploidy [81,164,208]	failed/asymmetrical cytokinesis, microcell, nuclear fusion, chromothripsis, aneuploidy
Horizontal DNA transfer	Yes (via microcell)	Yes (branching)
Retrovirus activation	Yes [165,209]	Yes [115]
Loss of Xist expression	Yes [210]	Yes [68]
OCT4/NANOG/SOX2 Expression	Yes	Yes [68]
Level of dedifferentiation	Complete	Complete or partial
Time of dedifferentiation	4–5 days	Variable, stress type and strength, cell type dependent
Out of dedifferentiation	Resume mitosis	Budding, splitting, viral burst, and horizontal genetic transfer, followed resumption of mitosis [113]
Next step in Development	Blastocyst (embryoblasts + trophoblasts)	Blastocyts-like structures [68] (embryoblast-like) + (trophoblasts?)
Three germ layers	Yes	Yes
Germ cells	Yes	Yes (germ cell tumor) [68]
Outcome	Fetus (+uterine implantation); teratoma and other tumors (−uterine implantation)	Teratoma and other tumors
Common tumor types	Immature tissue with no or minimal nuclear atypia; low grade tumor (Type 1 tumors) [21,201]	Marked nuclear atypia, anaplasia, PGCCs; high grade tumors (type II tumors) [21,201]
Tumor examples	Wilms’s tumor, immature teratoma, ependymoma, fibroma, small lymphocytic lymphoma [21,201]	High grade serous carcinoma, glioblastoma, triple negative breast cancer; anaplastic lymphoma [21,201]
